# Optical Coherence Tomography Angiography Predicts Visual Outcomes for Craniopharyngioma in Children by Quantifying Choroidal Capillaries

**DOI:** 10.3389/fmed.2021.819662

**Published:** 2022-01-12

**Authors:** Qing Lin Zhang, Jun Hua Wang, Li Ying Sun, Jian Bin Wang, Yu Ma, Yu Qi Zhang

**Affiliations:** Department of Neurosurgery, Tsinghua University Yuquan Hospital, Beijing, China

**Keywords:** craniopharyngioma, prognosis evaluation, optical coherence tomography angiography, children, quantifying choroidal capillaries

## Abstract

**Purpose:** To predict the prognosis of craniopharyngioma in children by optical coherence tomography angiography (OCTA).

**Methods:** We evaluated if the relationship between preoperative OCTA of the choroidal capillary density (CCD) and visual outcome continued over long-term visual recovery in 38 patients undergoing craniopharyngioma resection. Patients were evaluated 3 times: 1 week before surgery (Visit1), followed-up at 6–10 weeks (Visit2), and 9–15 months (Visit3) after surgery.

**Results:** In total 38 patients (70 eyes) with craniopharyngiomas, which included 20 (52.6%) boys and 18 (47.4%)girls, the mean age was 11.8 ± 2.7 years (range: 6–18 years). The age (*p* = 0.71), gender (*p* = 1.00), mean refractive error (*p* = 0.55), and axial length (*p* = 0.23) of 38 normal volunteers (76 eyes) were matched. After surgery, the cross-compression of patients was relieved. The average visual acuity change in the normal CCD group was 0.07 ± 0.02; the average visual acuity change in the low CCD group was 0.01 ± 0.01, *p* < 0.001. Preoperative CCD value is related to the preoperative BCVA (*p* < 0.001), and the visual function after the long-term follow-up (9–15 months) (*p* < 0.001). The prognosis of CCD has the same trend as the BCVA. Further correlation analysis shows that CCD and BCVA are significantly correlated (*r* = 0.878; *p* < 0.001). CCD has a weak but significant correlation both with MD (*r* = 0.19; *p* < 0.001) and PSD (*r* = −0.21; *p* <0.001). A natural cutoff of CCD is approximately 38%. With the normal CCD group the maximum improvement of BCVA exceeds 0.3 post-operatively, compared to eyes in the low CCD group that improve by <0.03, and worse after surgery.

**Conclusions:** Long-term vision recovery after surgical decompression of craniopharyngiomas in children can be predicted by preoperative by OCTA. Patients with normal CCD before surgery showed a tendency to improve vision; this trend of improvement persisted in subsequent follow-ups. The CCD baseline natural cutoff value for predicting visual prognosis before and after surgery is about 38%.

## Introduction

Craniopharyngioma, which develops from the remnant epithelial cells of the craniopharyngeal duct formed by the primitive ectoderm during the embryonic period, is the most common intracranial congenital tumor and has the second-highest incidence of all tumors in the sella area (1). It is known that 70% of craniopharyngioma occurs in children and adolescents under 15 years of age, and the annual population incidence rate is (0.5–2) per 1 million (1–3). The overwhelming majority of craniopharyngiomas affect visual acuity, and the prognosis of visual acuity after surgery is unpredictable. Besides, for patients with visually impaired craniopharyngioma, timely and effective surgical resection can restore vision in some patients, but there are still some patients with visual impairment (4). So there is an urgent need for accurate and feasible visual predictive quantitative indicators to determine the prognosis of patients' visual function before surgery, to assist the selection of surgical timing and strategies.

Researchers are committed to determining preoperative characteristic indicators to predict vision recovery for craniopharyngioma. Risk factors including age, duration of symptoms, tumor size and volume, preoperative visual acuity (VA) or visual field impairment (VF), optic nerve atrophy, and electroretinogram have all been studied, but none of them can accurately and consistently predict postoperative visual function for the limitations of subjectivity and difficulty in cooperation (5).

Recently, optical coherence tomography angiography (OCTA), as a new imaging technology using coherent light interference imaging, can non-invasively observe the blood vessel structure and blood perfusion *in vivo* in a short time (6). The instrument has been widely used in the ophthalmology field, and takes only 2–3 min to obtain anatomical tomographic images of the choroidal microstructure and perfusion quantitative indicator—choroidal capillary density (CCD)— which can comprehensively reflect the changes in visual acuity, and which has a good potential prognostic value may overcome the deficiencies in previous examinations to predicts visual outcome for craniopharyngioma in children (7–10).

In the pre-experimental study of case analysis, we found that in patients with cross-compressed craniopharyngioma, preoperative OCTA can predict postoperative visual acuity (VA) and visual field (VF) recovery. This study establishes a new quantitative indicator with OCTA to predict visual outcomes for craniopharyngioma in children by a prospective cohort study.

## Materials and Methods

### Participant Selection

Prospectively recruited patients diagnosed with craniopharyngioma from Yuquan Hospital of Tsinghua University from September 1, 2018, to January 1, 2021. This study follows the principles of the Declaration of Helsinki. The informed consent form was signed, and the study was approved by the ethics committee. Each patient is confirmed by MRI with cross-compression signs (may affect vision).

Inclusion criteria: for the experimental group, MRI confirmed that the patient's sellar area occupied space, and was independently clinically diagnosed as craniopharyngioma by two experienced neurosurgeons; the oppressed optic nerve will be treated by surgery; and receive postoperative follow-up. For the control group: volunteers with normal eyes with matching age, gender, and refractive state. All patients with craniopharyngioma need to undergo preoperative evaluation 1 week before surgery (Visit1), including VA, mydriatic fundus examination, visual field analyzer (Humphrey Field analyzer II; Carl Zeiss Meditec, Jena, Thuringia, Germany) and OCTA [(AngioVue^®^, Optovue Inc)., Fremont, California, USA)]. Follow-up examinations are performed 6 to 10 weeks (Visit2), and 9 to 15 months (Visit3) after surgery. The endpoint of the study is the finish of the second follow-up (Visit-3).

Exclusion criteria: anterior segment and posterior segment optic nerve diseases (except for compressive optic neuropathy), including glaucoma, cup-to-disk ratio asymmetry >0.2, focal optic disc notch, or optic nerve hemorrhage. Patients whose preoperative or postoperative VF test is unreliable (defined as 25% false positive, false negative, or fixed loss rate). Patients with high refractive errors [myopic or hyperopic refractive power (D) >5.0 D, astigmatism >2.0 D].

Thirty-eight patients (70 eyes) and 38 volunteers (76 eyes) were recruited. Six eyes were excluded based on the inclusion criteria, two patients had cup-to-disk ratio asymmetry >0.2; one eye preoperative VF test was unreliable; and three eyes had high refractive errors. All 38 operations were performed by the same surgeon (ZYQ). All patients underwent transcranial surgery to treat tumors. For the predictive follow-up analysis part, the patients were divided into two groups according to the ocular condition. One group is people with a normal average CCD (normal capillary perfusion), which is defined as the fifth percentile or higher of the age-matched standard value; the other group is people with a low average CCD, which is defined as below the fifth percentile of the age-matched standard value.

### Ophthalmic Examinations

The ophthalmic examination included measurement of best-corrected visual acuity (BCVA), slit lamp assisted biomicroscopy of the anterior segment of the eye, biometry applying optical low-coherence reflectometry (Lensstar 900^®^ Optical Biometer, Haag-Streit, 3098 Koeniz, Switzerland), and fundus photographs (non-stereoscopic photograph of 45° of the central fundus; fundus camera type CR6-45NM, Canon Inc. U.S.A.). Automatic refractometry (Auto Refractometer AR-610, Nidek Ltd, Tokyo, Japan) was performed on all the participants. If uncorrected visual acuity was 1.0 (i.e., 5/5), subjective refractometry was also performed. All the participants with undilated pupils were imaged with an OCTA (AngioVue^®^, Optovue Inc., Fremont, California, USA) with the instrument positioned close enough to the eye to produce a high-quality image. Using the Humphrey perimeter, the probability of pattern deviation image was used to record the mean deviation (MD) and standard deviation (PSD) of the model before and after the operation (Visit1, Visit2, Visit3). MD reflects the decrease in average visual acuity caused by various factors, which can come from damage to the optic nerve or even from the refractive interstitial opacity, which is around 0 dB in normal people; while PSD can filter out those decreased visual acuity caused by the refractive interstitium. The large absolute value of the two indices represents the loss of overall or partial vision, respectively.

### Brain Imaging

Patients underwent MRI neuroimaging before surgery (Visit1), 6–10 weeks after surgery (Visit2), and 9–15 months after surgery (Visit3). The scale designed by Fujimoto et al. was used to analyze the coronal position T1-weighted imaging to score optic chiasm decompression (11). Grade 0: The tumor has no contact with the optic nerve. Grade 1: The tumor is in contact with the optic chiasm but there is no notch or deformation on the cross surface. Grade 2: The tumor compresses the optic chiasm and causes deformation but the superior optic chiasm is visible. Grade 3: The tumor compresses the optic chiasm. Deformation is not visible in the suprachiasmatic cistern. Grade 4: The tumor causes severe optic chiasm compression and brain malformations. The size of the tumor was measured before surgery, and the decompression grade assessed after surgery.

### Measurement of CCD

**Figure 1:**
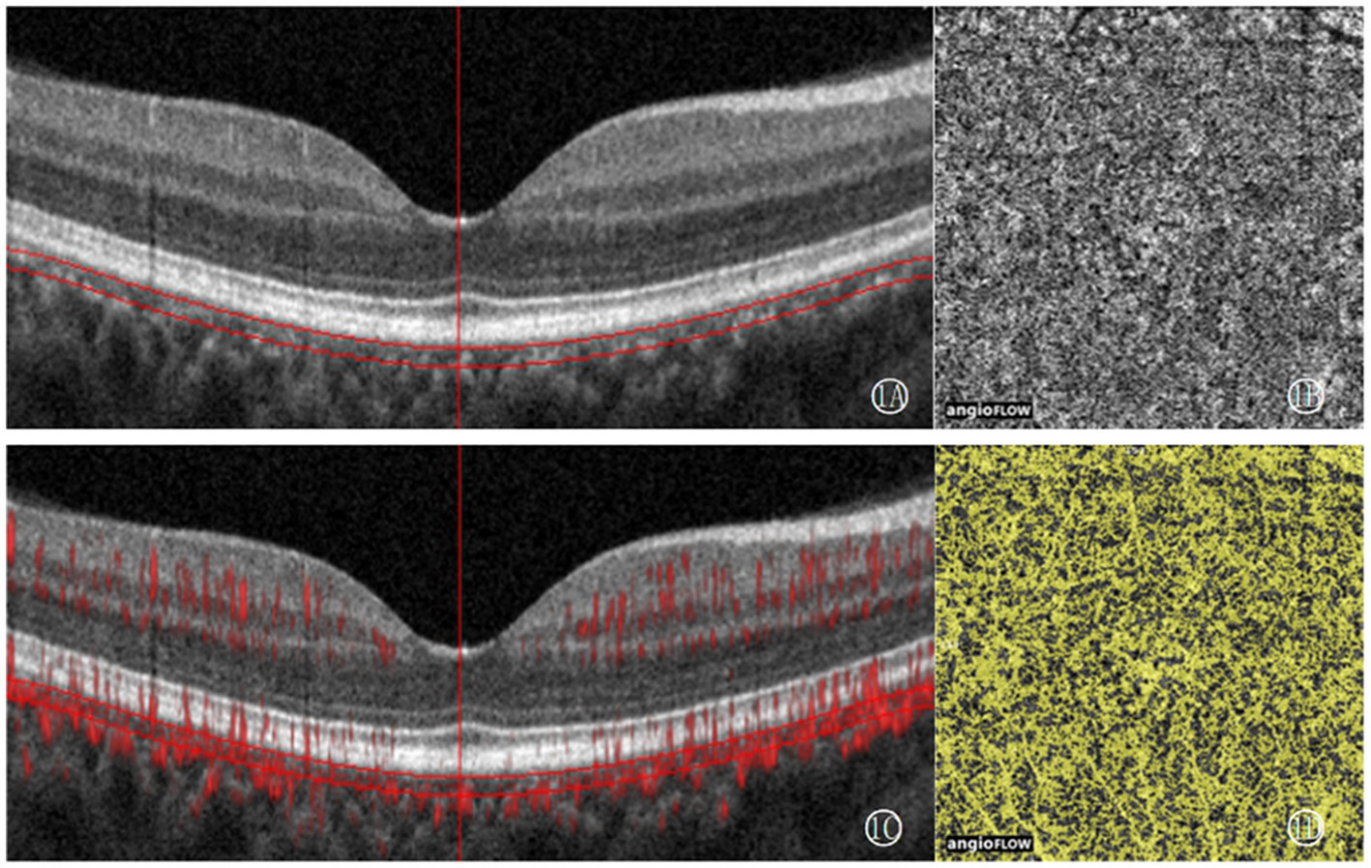
CCD measurement figures: select the choroidal capillary layer, use OCTA vascular quantification 2.0 software to measure the density of the choroidal capillary layer in 3 × 3 mm mode, that is, the total area of blood flow divided by the total area of the layer (9 mm^2^) ×100%. **(1A)** shows the defined measurement level, between the parallel lines is the choroidal capillary layer, the vertical line is the macular fovea position calibration line, **(1B)** shows the en-face image, and **(1C)** shows the blood flow signal acquisition image. **(1D)** shows the blood flow pixel image.

OCTA is used to follow-up and analyze the distribution of choroidal capillaries in patients of craniopharyngioma. An image with a size of 3 × 3 mm centered on the macular arch was collected, and the CCD was quantitatively measured by OCTA vascular quantification 2.0 software (ReVue XR, version 2017.200.0.35; Optovue Inc). The choroidal capillary layer is from the reference plane of the retinal pigment epithelium (30 μm) to the reference plane of the large and medium choroidal blood vessels (6). CCD is defined as the area of the choroidal capillary blood flow pixel (yellow) in the 3 × 3 mm mode and the total area of the layer (9 mm^2^) × 100% (Figure 1). All operations were completed by the same experienced examiner (QSQ), and all OCTA images with poor imaging quality were excluded. The image feature analysis and quantitative measurement of all images were independently completed by three experienced ophthalmologists.

### Statistical Analyses

SPSS statistical software package for statistical analysis (SPSS for Windows, version 22.0. SPSS, Chicago, IL, USA). All measurement data are presented as the mean ± standard deviation. The eyes were grouped into low CCD and normal CCD groups based on their choroidal blood perfusion. Grouping is used as an independent factor in the analysis. Independent sample *t*-test to analyze the significant difference of variables in baseline (Visit1) and changes of short-term (Visit2–Visit1) and long-term (Visit3–Visit1) between the low CCD and normal CCD groups. Regression analysis evaluates the correlation between postoperative CCD and BCVA/VF. Comparison of the magnitude of BCVA change from pre-operative to post-operative assessment among eyes in the low and normal CCD group and evaluate a natural cutoff point by extreme value method. All *P*-values are < 0.05 using a two-sided test.

## Results

### Preoperative Baseline Data

In total there were 38 patients (70 eyes) with craniopharyngiomas, which included 20 (52.6%) boys and 18 (47.4%) girls, the mean age was 11.8 ± 2.7 years (range: 6–18 years). The age and gender of 38 volunteers (76 eyes) normal volunteers were matched (*p* all >0.05), which the mean age was 11.6 ± 2.8 years old (*p* = 0.71) (range: 6–18 years) (Table 1). There was no significant statistical differences in the mean refractive error (*p* = 0.55) and axial length (*p* = 0.23) in the craniopharyngioma and control group. However, the mean BCVA of patients (0.55 ± 0.34) was supposedly worse than normal volunteers (1.01 ± 0.19, *p* < 0.001).

**Table 1:** Comparison of general clinical data between craniopharyngioma group and normal control group ($\bar{x}$ ± s).

	**Craniopharyngioma group**	**Normal control group**	***P*** **-value**
Age	11.8 ± 2.7	11.6 ± 2.8	0.71
Gender (Male/Female)	20/18	20/18	1.00
Refractive error (D)	0.08 ± 0.41	0.10 ± 0.37	0.55
Axial length (mm)	23.62 ± 1.43	23.48 ± 0.21	0.37
BCVA	0.55 ± 0.34	1.01 ± 0.19	<0.01

### Postoperative Changes in Visual Function

The preoperative craniopharyngioma size was 3.5 ± 1.2 cm in the normal CCD group, and showed no statistical difference compared to the low CCD group (3.4 ± 1.4 cm, *p* = 0.742). After surgery, the cross-compression of patients was relieved, and 84.2% (32 cases) had MRI scores of Grade 0, 10.5% (4 cases) of Grade 1, and 5.2% (2 cases) of Grade 2. The low CCD group, defined as below the fifth percentile of the age-matched standard value, included 29 (41.4%) eyes; the normal CCD group, defined as the fifth percentile or higher of the age-matched standard value, involved 41 eyes (58.6%). Among the eyes of the low CCD group, 75.9% (22 eyes) were Grade 0 patients, 13.8% (4 eyes) were Grade 1 patients, and 10.3% (3 eyes) were Grade 2 patients. Whereas, 90.2% (37 eyes) with normal CCD were Grade 0 patients, 4.9% (2 eyes) were Grade 1 patients, and 4.9% (2 eyes) were Grade 2 patients. There is no statistical difference in grading between the two groups (*p* = 0.672) (Table 2).

**Table 2:** Comparison of the visual prognosis of the low CCD group and the normal CCD group after decompression.

**Variable**	**Visit1: baseline**		**Visit2: postoperative** **2-6 weeks**		**Visit3: postoperative** **9–15 months**		**P1**	**P2**	**P3**	**P4**
**Group**	**Normal**	**Low CCD**	**Normal**	**Low CCD**	**Normal**	**Low CCD**				
Preoperative craniopharyngioma size (cm)	3.5 ± 1.2	3.4 ± 1.4					0.742			
Postoperative decompression grade (0/1/2)	37/2/2	22/4/3					0.672			
BCVA	0.58 ± 0.29	0.21 ± 0.16	0.57 ± 0.24	0.18 ± 0.09	0.65 ± 0.24	0.22 ± 0.11		0.642	0.036	0.039
MD	−4.6 ± 1.1	−9.9 ± 2.3	−2.2 ± 0.8	−7.3 ± 2.2	−0.9 ± 0.2	−3.5 ± 1.2		<0.001	0.001	0.014
PSD	4.7 ± 0.9	8.7 ± 1.3	3.2 ± 0.3	6.2 ± 0.8	2.1 ± 0.2	4.5 ± 0.6		0.012	0.003	0.006
CCD	47.9 ± 3.3	23.4 ± 2.1	47.3 ± 3.1	22.9 ± 1.9	58.6 ± 3.4	27.4 ± 2.2		0.792	<0.001	<0.001

*P1: Differences between Normal and Low CCD group in baseline (Visit1)*.

*P2: Differences of changes (Visit2–Visit1) from visit 2 to 1 between Normal and Low CCD group*.

*P3: Differences of changes (Visit3–Visit1) from visit 3 to 1 between Normal and Low CCD group*.

*P4: Differences of changes (Visit3–Visit2) from visit 3 to 2 between Normal and Low CCD group*.

Further comparison of the visual prognosis of the low CCD group and the normal CCD group was carriad out after decompression. For the short-term visual acuity after the operation (2–6 weeks, Visit2), the normal CCD group and the low CCD group had no significant changes (*p* = 0.642); but for the long-term visual acuity after the operation (9–15 months, Visit3), the normal CCD group improved significantly more than the low CCD group (*p* = 0.036). The short-term (2–6 weeks, Visit2) and long-term (9–15 months, Visit3) visual field (MD and PSD) after operation were significantly reduced in the normal CCD group and the low CCD group (*p* all < 0.05). In the short-term postoperative (2–6 weeks, Visit2), there was no significant change in CCD of the normal and low CCD groups (*p* = 0.792); but postoperative long-term (9–15 months, Visit3), the normal CCD group improved significantly than the low CCD group (*p* < 0.001) (Table 2).

**Figure 2:**
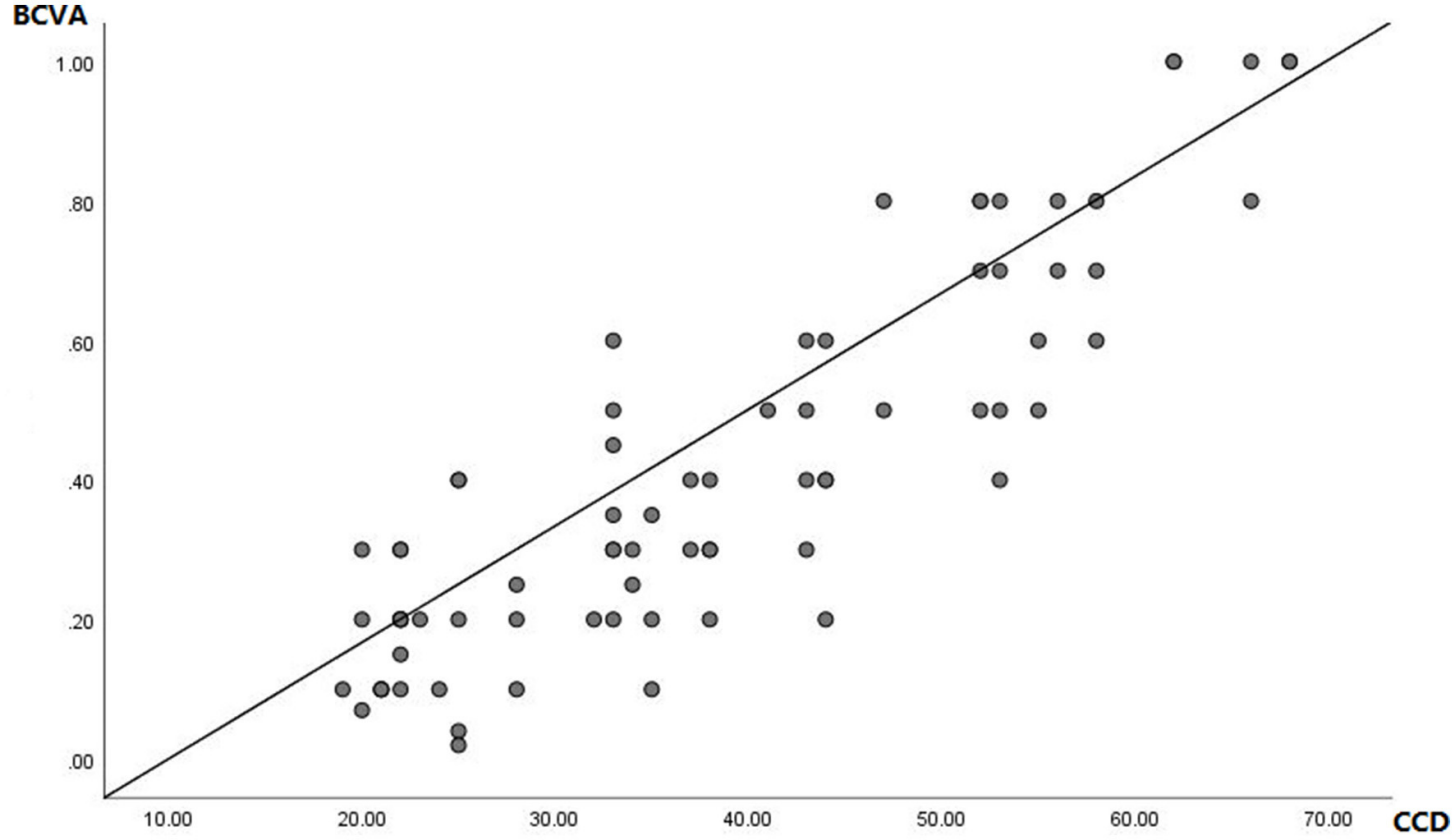
Linear regression image comparing the prognosis changes of CCD and BCVA. *y* = 0.0167* *x* + −0.1667, *r* = 0.878; *p* < 0.001.

The prognosis of CCD has the same trend as the BCVA. Further correlation analysis shows that CCD and BCVA are significantly correlated (Figure 2; *y* = 0.0167 ^*^ *x* + −0.1667, *r* = 0.878; *p* < 0.001). Besides, CCD has a weak but significant correlation both with MD (*r* = 0.19; *p* < 0.001) and PSD (*r* = −0.21; *p* < 0.001).

The preoperative (Visit1) and long-term (Visit3) visual acuity differences between the normal and low CCD groups were significant and parallel to CCD. We further analyzed the long-term visual acuity recovery (BCVA of Visit3–Visit1) and the CCD baseline before and after different groups of operations. The average visual acuity change was 0.07 ± 0.02 in the normal CCD group; and 0.01 ± 0.01 in the low CCD group (*p* < 0.001).

**Figure 3:**
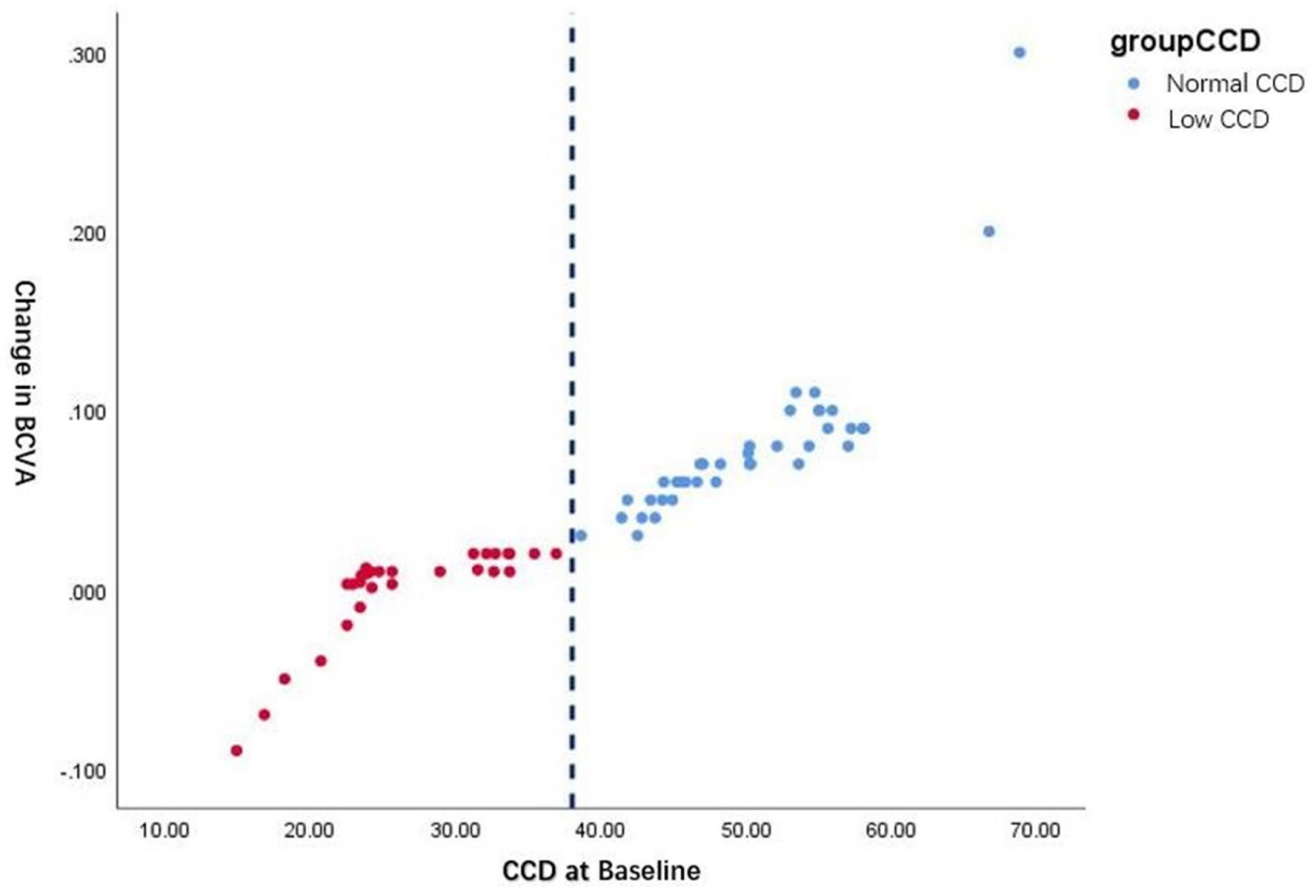
Comparison of the BCVA changes from pre-operative to post-operative assessment among eyes in the low and normal CCD group.

Figure 3 shows the natural cutoff of CCD is ~38%. With the normal CCD group the maximum improvement of BCVA exceeds 0.3 post-operatively, compared to eyes in the low CCD group that improved by <0.03, even became worse after surgery than they were before.

## Discussion

As the most common symptom in patients with craniopharyngioma, especially in children, the prognosis of visual function is always an important focus (12). In previous clinical practice, subjective examinations such as visual acuity and visual field were mostly used, which have a learning curve, but the need to repeat the measurement many times (13, 14). Objective examinations such as electroretinogram and optic nerve atrophy images are comprehensive indicators of the entire eye, and mainly target nerve tissue. These do not reflect the early damage of the cross-compression area and do not consider the very early microcirculation changes caused by blood supply disorders, and so are difficult to use as a quantitative indicator of visual prognosis (5, 6, 14). In addition, the above-mentioned examinations are difficult to operate, with long examination times and high coordination requirements. It is difficult for patients to complete the examination in the early postoperative period, especially for children (12).

This study found that long-term vision recovery after surgical decompression of craniopharyngiomas in children can be predicted by preoperative OCTA, which is expected to become a new prognostic indicator of visual acuity (7–10). The new CCD index established in this study reflects choroidal microcirculation in real-time. Patients with a severe reduction in CCD indicate blood supply disorder of the ocular visual center before surgery (15). The results show that although most patients have an improvement to a certain extent of visual function (BCVA, MD, PSD) after surgery. However, patients with low CCD have relatively poor visual acuity and visual field recovery. The average visual acuity change in the normal CCD group was 0.07 ± 0.02; the average visual acuity change in the low CCD group was 0.01 ± 0.01, *p* < 0.001. Otherwise, it reveals that preoperative CCD value is not only related to the preoperative BCVA but also associated with the visual function after the long-term follow-up (9–15 months) (*p* all < 0.05).

Many studies (16–19) can explain the above results. Previous research has found that in patients with sellar tumors that cause compression of the optic nerve, the reasons for the visual impairment may be divided into the following three types. First, the space-occupying tumor physically compresses the optic nerve and causes nerve deformation and mechanical damage to an excessive extent (1, 4, 5). MRI-based imaging studies revealed that the severity of visual acuity and visual field damage is related to the volume and location of the tumor, and also the degree of compression of the optic nerve (5). However, some patients with insignificant nerve compression will still have severe visual impairment after the compression is released in time (2). Therefore, the pure mechanical injury hypothesis cannot support the evaluation of visual prognosis; but confirms the results of the recent study. Even if most of the mechanical compression is completely relieved, which in the 84.2% with Fujimoto Grade 0 and 1 after the operation in our research it was, the visual prognosis may be improved, the same, or worse. Second, microcirculation ischemia of the ocular visual center by the decrease in blood supply. If the ischemia causes damage to the visual cells, even if the mechanical pressure is quickly removed, the visual function cannot be improved (20). This similarly confirms the results of the recent study. The visual prognosis of patients without choroidal microcirculation disorder (Normal CCD group) before surgery is significantly better than that of the ischemic group (Low CCD group). Third, it is a combination of the two factors, which was the widely accepted hypothesis. In summary, for patients with craniopharyngioma, the evaluation of the visual prognosis requires comprehensive analysis. Previous brain imaging indicators such as MRI can be used to assess the degree of mechanical damage before surgery, while CCD may become the new index of microcirculation disorder in early prediction.

Furthermore, the results show that the natural cutoff of CCD is ~38%, which indicates that children with optic chiasm compression craniopharyngioma whose preoperative CCD is lower than 38 have poor visual prognosis after surgery; and in children with a preoperative CCD >38, visual acuity may be significantly improved after surgery. Besides, the improvement is parallel to preoperative CCD.

This is similar to a study about the recovery of visual acuity of patients with pituitary tumors, which revealed that better preoperative visual acuity was associated with visual function improvement (5). Similar to our results, it showed the improvement of BCVA and visual field is not obvious in the early postoperative period, and even some patients will get worse; but long-term follow-up can find significant recovery (21). This may be due to the ischemia-reperfusion injury or optic nerve injury due to decreased intracranial pressure after removal of the tumor (22, 23). Overall, the long-term prognosis is the appropriate time point for visual acuity assessed by CCD before surgery.

Potential limitations should be mentioned. First, differences between participants and non-participants may have led to a selection artifact with a hospital-based study. Second, CCD does not represent the recovery of the patient's visual field, which may indicate that CCD only represents the visual acuity of the fovea and cannot represent the peripheral visual function. Third, due to the impact of the COVID-19 epidemic, timely follow-up was affected. Although the sample size is sufficient but still small, the sample size needs to be further increased in future study to clarify the predictive value of the CCD for peripheral vision function. Third, because the scanning rate, laser wavelength, and algorithm could be different between various OCTA devices, the cutoff point in different OCTA devices may be different. The consistency of detection of different types of instruments should be further studied in the future.

In conclusion, long-term vision recovery after surgical decompression of craniopharyngiomas in children can be predicted by preoperative by OCTA. Patients with normal CCD before surgery showed a tendency to improve vision; this trend of improvement persisted in subsequent follow-ups. The long-term and short-term vision recovery of patients with preoperative low CCD is not obvious, and the vision will be temporarily reduced in the short term (2–6 weeks) after the operation. The long-term vision prognosis may be similar to that before the operation, and there is no significant improvement. This study found that the CCD baseline natural cutoff value for predicting visual prognosis before and after surgery is about 38%. That is, in children with a preoperative CCD >38%, visual acuity may be significantly improved after surgery, and the degree of postoperative improvement parallel to the CCD before surgery.

## Data Availability Statement

The original contributions presented in the study are included in the article/supplementary material, further inquiries can be directed to the corresponding author/s.

## Ethics Statement

All procedures followed were in accordance with the ethical standards of the responsible committee on human experimentation (institutional and national) and with the Helsinki Declaration of 1975, as revised in 2000 and 2008. Informed consent was obtained from all patients for being included in the study.

## Author Contributions

QZ designed the study and wrote the initial draft of the paper. YZ was in charge of administration and management of the study. All authors contributed to collecting and analyzing the data and were involved in writing the manuscript and approved the revision and the submission.

## Conflict of Interest

The authors declare that the research was conducted in the absence of any commercial or financial relationships that could be construed as a potential conflict of interest.

## Publisher's Note

All claims expressed in this article are solely those of the authors and do not necessarily represent those of their affiliated organizations, or those of the publisher, the editors and the reviewers. Any product that may be evaluated in this article, or claim that may be made by its manufacturer, is not guaranteed or endorsed by the publisher.
